# Control of Biological Hazards in Insect Processing: Application of HACCP Method for Yellow Mealworm (*Tenebrio molitor*) Powders

**DOI:** 10.3390/foods9111528

**Published:** 2020-10-24

**Authors:** Pauline Kooh, Vanessa Jury, Sophie Laurent, Frédérique Audiat-Perrin, Moez Sanaa, Vincent Tesson, Michel Federighi, Géraldine Boué

**Affiliations:** 1 French Agency for Food, Environmental and Occupational Health & Safety, Risk Assessment Department, 14 rue Pierre et Marie Curie, 94701 Maisons-Alfort, France; pauline.kooh@anses.fr (P.K.); frederique.audiatperrin@anses.fr (F.A.-P.); moez.sanaa@anses.fr (M.S.); 2 Oniris, Université de Nantes, CNRS, GEPEA, UMR 6144 F-44000 Nantes, France; vanessa.jury@oniris-nantes.fr (V.J.); sophie.laurent@oniris-nantes.fr (S.L.); 3 INRAe, Oniris, Secalim UMR 1014, route de Gachet, CS 40706, 44307 Nantes, France; vincent.tesson@inrae.fr (V.T.); michel.federighi@oniris-nantes.fr (M.F.)

**Keywords:** entomophagy, *Tenebrio molitor*, insect powder, edible insects, microbial hazards, food safety, HACCP, risk assessment, predictive microbiology, insects processing, novel food

## Abstract

Entomophagy has been part of human diets for a long time in a significant part of the world, but insects are considered to be a novel food everywhere else. It would appear to be a strategic alternative in the future of human diet to face the challenge of ensuring food security for a growing world population, using more environmentally sustainable production systems than those required for the rearing of other animals. *Tenebrio molitor*, called yellow mealworm, is one of the most interesting insect species in view of mass rearing, and can be processed into a powder that ensures a long shelf life for its use in many potential products. When considering insects as food or feed, it is necessary to guarantee their safety. Therefore, manufacturers must implement a Hazard Analysis Critical Control plan (HACCP), to limit risks for consumers’ health. The aim of this case study was to develop a HACCP plan for *Tenebrio molitor* larvae powders for food in a risk-based approach to support their implementation in industry. Specific purposes were to identify related significant biological hazards and to assess the efficiency of different manufacturing process steps when used as Critical Control Points. Then, combinations of four different processes with four potential uses of powders by consumers in burger, protein shake, baby porridge, and biscuits were analyzed with regard to their safety.

## 1. Introduction

The provision of traditional animal protein will require more agricultural land for crops to feed livestock than will be available, given the increase in the world population [1,2]. In addition, increases in livestock numbers are associated with negative environmental impacts. Therefore, there is a real need for production of alternative protein sources. Proteins of vegetable origin were identified as the first candidates and have been thoroughly investigated as they are already present in the human diet [3,4]. Another alternative is proteins from insect sources, as highlighted in several agency reports and scientific communications [5,6]. The rearing of insects is expected to have lower adverse environmental impact as it requires less agricultural land and consumes less water [3,4,7]. Consequently, all around the world, the mass rearing of edible insects is attracting growing interest, especially with regard to three species: *Hermetia illucens*, the black soldier fly, *Tenebrio molitor*, the yellow mealworm, and *Acheta domesticus*, the house cricket. Traditional and innovative technologies have been used to process insects and have provided two predominant forms: whole insects or powder [8]. The consumption of these insects with their digestive tract is necessarily a vector of numerous microbiological hazards with high levels of contamination [9]. Moreover, insects can provide a rich environment for microbial survival and growth, due to their high nutrient content and high water activity [9].

According to the European Union (EU) general food law, food placed on the market must be safe, i.e., food (including Novel Food) must not be injurious to health or unfit for human consumption [10]. The general system designed to control the safety of human and animal food has undergone profound changes and modifications for several decades inspired by Codex Alimentarius works, in particular, on the Hazard Analysis and Critical Control Points method (HACCP). At the same time, the use of this method has spread widely in food industries and in national and international regulations. In the EU, the major evolution came about with the general food law in 2002, and other “hygiene package” legislation, which establishes the coordinated and integrated general framework for all food business operators (primary producers, manufacturers, retailers, etc.) [10]. Member States have therefore developed a system that requires that “no hazardous foods be placed on the market” (Article 14), the rules in force apply from “farm to fork” and are determined following a risk analysis procedure. Food chain operators/actors are responsible for the safety of the food they produce, and they have to use principles of the HACCP method to build their food safety plan as well as to apply the hygiene rules in force and to control the potential hazards of their sector of activity. This must be considered in the design, organization, and management of food production sites and the quality assurance label for an activity, the hygienic design of premises and equipment, the development of a product traceability system, and the implementation of pre-requisite programs such as Good Manufacturing Practices (GMPs) and Good Hygienic Practices (GHPs) [11]. GHPs are defined as the basic conditions and activities necessary to maintain a hygienic environment suitable for the production, handling, and availability of safe end products and safe food for human consumption throughout the food chain. These GHPs are essential for the implementation of a HACCP plan. It relies on the European Commission (EC) Regulation 852/2004 on the hygiene of foodstuffs (OJ L 139, 30.4.2004), the EC Regulation 853/2004 on specific hygiene rules for food of animal origin (OJ L 139, 30.4.2004), and the EC Regulation 183/2005 on requirements for feed hygiene (OJ L 35, 8.2.2005) [12,13,14].

The HACCP is a method structured by 7 principles and 12 tasks allowing to guard against all issues related to food safety through the implementation of operational activities, means, and pre-established technical solutions and to provide proof, as recommended in CAC/RCP-1 1969, rev. 4 2003 [15]. The implementation of the HACCP method in insect processing industries is thus mandatory and will enable the strengthening of the safety of delivered end-products. However, as this industry is emerging, there is still little to guide its implementation. To date, we have identified the International Platform of Insects for Food and Feed (IPIFF) [16] guide, which explains the general principles of the HACCP method and gives a few examples by way of illustration, but its purpose is more educational than applicative. Also, Fraqueza and Patarata [17] have highlighted that HACCP implementation is a key element in further developing this industry, as one of the main limitations involves guaranteeing the safety of the product. They have reviewed available information for different insect species and have highlighted associated constraints. They are mainly related to the hazard analysis that is limited by the lack of contamination and epidemiological data, as well as the validation of control measures and critical limits as pathogens’ behavior could be different in insects.

To progress further on this subject, the present paper develops, for biological hazards, a HACCP plan for an operational case study: the production of yellow mealworm (*Tenebrio molitor*) powders for food. This insect species was selected as it has a fast and well-controlled rearing method, is already used for feed and food in many countries, and has a high marketability potential [18]. The case study focuses on the manufacturing of insect in powder form as this has a greater potential for inclusion into Western countries’ diets than whole insects. It has a long shelf life, it can be mixed with many other ingredients and thus included in many recipes, it has a mild flavor, aroma, and color compared to whole insects, and so this would be a good way to include these products without insects being visible, which could improve acceptability [19]. Specific purposes were to identify related significant biological hazards and to assess the efficiency of different manufacturing process steps to act as Critical Control Points. Then, in a risk-based approach, the safety of the combinations of four different processes was assessed, with four potential uses of powders by consumers (in burger, protein shake, baby porridge, and biscuits).

## 2. Definition of the HACCP Application

**Figure 1:**
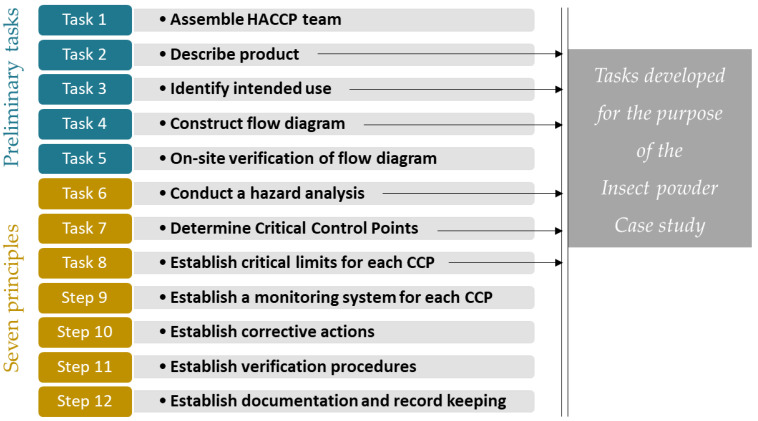
Schema of Hazard Analysis and Critical Control Points (HACCP) tasks and overview of the main elements developed in the case study.

The present project develops a HACCP plan for biological hazards in mealworm powders. The HACCP plan was developed following the twelve steps of the method (Figure 1). First, the HACCP team was assembled (Task 1), gathering multidisciplinary expertise in mass rearing and processing of insects, biological hazards, modelling, and risk analysis, as well as experience in the HACCP method (with theoretical and practical knowledge). Then, a specific emphasis was provided for task 2 (product description) and task 3 (identification of use), to refine our case study. Moreover, task 6 of hazard analysis has been thoroughly researched, as significant biological hazards are not yet clearly defined for this novel food. A transparent and documented method has been applied. Then, Critical Control Points were determined (Task 7) and critical limits were investigated using a quantitative and risk-based approach.

### 2.1. Description of Tenebrio molitor Powder Products

*Tenebrio molitor* powders are described in Table 1, including information on raw materials and a description of the end product, providing its chemical constituents, product characteristics, packaging, destination, labeling information, shelf life, and storage conditions. The description includes the two common categories of mealworm powders, that is, whole (full-fat) mealworm powder and defatted mealworm powder.

### 2.2. Definition of the Potential Use of Tenebrio molitor Powder

**Figure 2:**
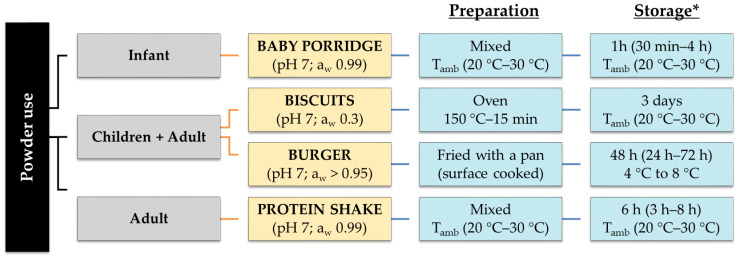
Description of four potential uses of *Tenebrio molitor* powders. * In the absence of data, ranges of storage conditions were considered to reflect potential variability of uses. For instance, 1 h (30 min–4 h) means that the storage duration was considered around 1 h but could vary between 30 min and 4 h. Similarly, T_amb_ (20–30 °C) stands for a temperature varying from 20 to 30 °C.

Insect powder can be used as an ingredient in a long list of potential recipes including snacks, crackers, pastries, cookies, candy, chocolate, paste, noodles, breads, sausages, meat loaves, as well as special dishes including burgers [26,27]. To date, insect products are not included in national dietary surveys in Europe and are not consumed in sufficient volumes to provide a clear view of their potential uses. To investigate different cooking and storage practices, we defined four different potential uses representing key models of possible situations and considered different targeted populations (Figure 2). This included use in baby porridge, already a focus of interest in Cambodia [28] and that appears to be a strategy for combating undernutrition by increasing protein content. Protein shake corresponds to a current trend among the athletes aimed at increasing the proportion of protein in their diet to develop muscle mass, a particular form of use with a high consumption potential in terms of market share. Biscuits were selected because many of the innovative products containing insects in Western countries are savory or sweet cookies. Finally, vegetable burgers represent a direct substitution alternative to beef. The term “burger” will refer to the substitute of ground beef with a mixture of insects and additional ingredients, as currently found in soy steaks, for instance.

### 2.3. Identification of Possible Tenebrio molitor Powder Manufacturing Process

**Figure 3:**
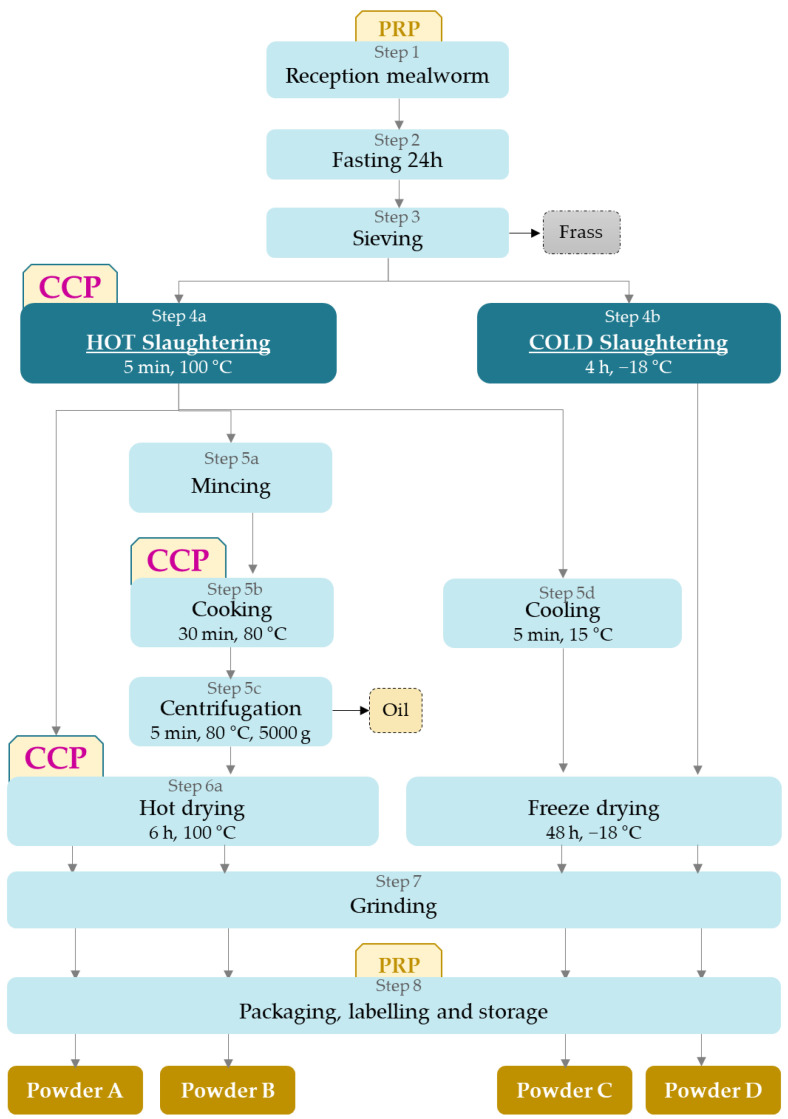
Description of four *Tenebrio molitor* powder manufacturing processes.

Raw materials are collected alive at the end of the *Tenebrio molitor* larvae stage (before the pupa stage). They are reared following good manufacturing practices: in a closed and controlled environment, and are fed on dry substrates, mainly cereal-based materials supplemented with fruits and vegetables or/and their derivatives. *Tenebrio molitor* larvae are collected from containers or chambers at the larval stage of their cycle [16]. Larvae live in their substrate and frass. They undergo 24 h of fasting to clear the gastrointestinal tract and then larvae are sieved to remove feces and substrate [16]. To date, the efficiency of fasting is not demonstrated and would need to be investigated to see if this step is required. Then, the manufacturing process can follow different strategies [8,26]. In the present case study, four options were selected to investigate different possible impacts on end-product safety (Figure 3). Parameters were determined from a recent patent [22], based on current industrial practices for process B, while processes A, C, and D were included as they correspond to current on-going research interests. After fasting and sieving, the live whole yellow mealworms are (1) blanched, dried, and ground, or (2) blanched, defatted, dried, and ground, or (3) blanched, freeze-dried, and ground, or lastly, (4) frozen, freeze-dried, and ground.

The manufacturing steps are described in Table 2. The combination of these four processes with the four potential uses will provide an assessment of 16 potential scenarios. Two processes for the slaughtering of insects are used. The first one is hot slaughtering in boiling water for 5 min. The boiling step can be done in a thermostatically controlled double-walled tank with stirring, with an insect:water ratio of 1:1. The tank is equipped with a temperature sensor with a continuous record of temperature and the 5-min period only starts when the temperature reaches 100 °C.

Then, the drained insects are dried in a fine layer in a hot air-dryer at 100 °C during 6 h. Insects are then ground and packaged to produce the powder A. An alternative to this process is that after hot slaughter, drained mealworms are minced and then cooked with a ratio insect:water of 1:0.5 during 30 min at 80 °C in a thermostatically controlled double-walled tank with stirring, centrifuged to separate oil and paste. Next, this insect paste is returned to the previous transformation process with drying, leading to powder B. Another branch of the process after hot slaughtering is to cool insects during 5 min at 15 °C and to freeze-dry the whole insects. Freeze-dried insects are then ground, leading to the production of powder C.

The other option for slaughtering is cold slaughtering which consists of freezing whole insects during 4 h at −18 °C. After a storage step, insects undergo freeze-drying. This option leads to powder D. For all processes, the drying phase is stopped only when the water activity is below 0.5.

## 3. What Are the Main Hazards of Concern? Analysis and Selection of Potential and Significant Hazards

A central pillar of any HACCP plan is the hazard analysis (task 6). First, the HACCP team has to identify all hazards that may be reasonably expected (named potential hazards), as a “long list” [29], to occur in the product, i.e., the four considered for *Tenebrio molitor* powders. This identification must consider raw materials, inputs and ingredients used (including rearing substrate in our case), the overall production process implemented on-site, the expected conditions of use by the end-user or the consumer, as well as the reasonably expected misuses.

### 3.1. Identification of a “Long List” of Reasonably Expected Biological Hazards

According to the description of a few authors on the microbial characteristics of edible insects, it seems that the intrinsic natural microflora of insects is different from that of humans or warm-blooded animals. For example, probably because of the species barrier, entomopathogenic microorganisms belong to very different phyla from human foodborne pathogens [5,18,30,31]. More recently, the microbiological safety of edible insects has been of concern in literature reviews and agencies’ opinions [30,32,33,34]. It appears from these reviews that prions are not a relevant hazard for edible insects. Similarly, it is reasonable to assume that major foodborne parasites are not potential hazards for the edible insects sector [32]. Several authors also assume that yeasts and molds are not potential hazards of concern for human health [30,32]. However, mycotoxins, chemical hazards of biological origin, are relevant potential hazards for *Tenebrio molitor* when reared on a cereal substrate [35]. As chemical hazards, mycotoxins are not in the scope of this case study.

Finally, a “long list” of potential microbiological hazards in edible insects was established, based on previous studies [30,32,33,34] (Table 3). This long list was then analyzed to identify the ones that have to be controlled (i.e., eliminated or sufficiently reduced) to ensure the safety of the product. Main reservoirs of each hazard are identified in Table 3. The contamination of insects by these hazards may originate from the substrates, the processing environment, or human operators. These hazards can persist throughout the process, with varying levels of microorganism sensitivity according to their individual characteristics.

### 3.2. Hazard Analysis

General methodology: Different tools (rating grids, FMEA grids (Failure Mode Effect Analysis), two or three dimension risk matrix…) can be used to select a list of significant hazards from the list of potential microbiological hazards. These tools have a point in common, they all take into account the likelihood and the severity of adverse effects, an important dimension in a public health context. In this case study, we established a risk score for every potential hazard listed in Table 3, based on the Severity and Likelihood index.

Severity index: The severity was expressed as the DALY (Disability Adjusted Life Year) per case. We used a numerical scale (1, 3, and 5) to derive the DALY of the diseases [36,37]. Hazards associated with a high severity received the maximum score (5) and those associated with low severity have the minimum score (1). The DALY estimates were collected in different reports [37,38,39,40]. It has to be noted that the severity associated with Shiga-toxin-producing *E. coli* was due to Hemolytic uremic syndrome (HUS).

Likelihood index: The second dimension is a likelihood index based on two criteria:

- The relevance of the hazard reservoir regarding its potential presence in edible insects, taking account of breeding and the four different manufacturing processes of *Tenebrio molitor* powder: this criterion is named Reservoir (R) in Table 4. The score for the relevance of the reservoir is 5 for telluric microorganisms, 3 for ubiquitous microorganisms, and 1 for microorganisms with a very specific reservoir, such as birds, animals of the *Suidae* family, or humans.
- The capacity of the hazard to survive and persist during the breeding, the processing, and the storage of *Tenebrio molitor* powders: this criterion is named Persistence (P) in Table 4. According to the type of processing flow chart, there are two scores of persistence.
  - For powders A, B, and C, the score of persistence is 5 for sporulated microorganisms, the distribution of the other microorganisms between the scores of 3 or 1 is based on the known resistance of the microorganism outside their natural reservoir and their resistance to the different processing steps of production of *Tenebrio molitor* powder A, B, and C, namely hot thermal treatments. In addition, microorganisms reputed to be thermosensitive, like *Campylobacter* spp., have a reduced score of 1.
  - For Powder D, the persistence score is adjusted upwards for Hepatitis A virus, Norovirus, Histamine, *Campylobacter* spp., and *Yersinia* spp., as there is no thermal treatment in the manufacturing process.

Finally, the Likelihood index (Li) corresponds to the product of the two criteria, i.e., Reservoir and Persistence (Li = R × P).

Risk score: The Risk score is obtained by the multiplication of Likelihood index and Severity score (Risk = Li × S). These scores are presented in Table 4.

**Figure 4:**
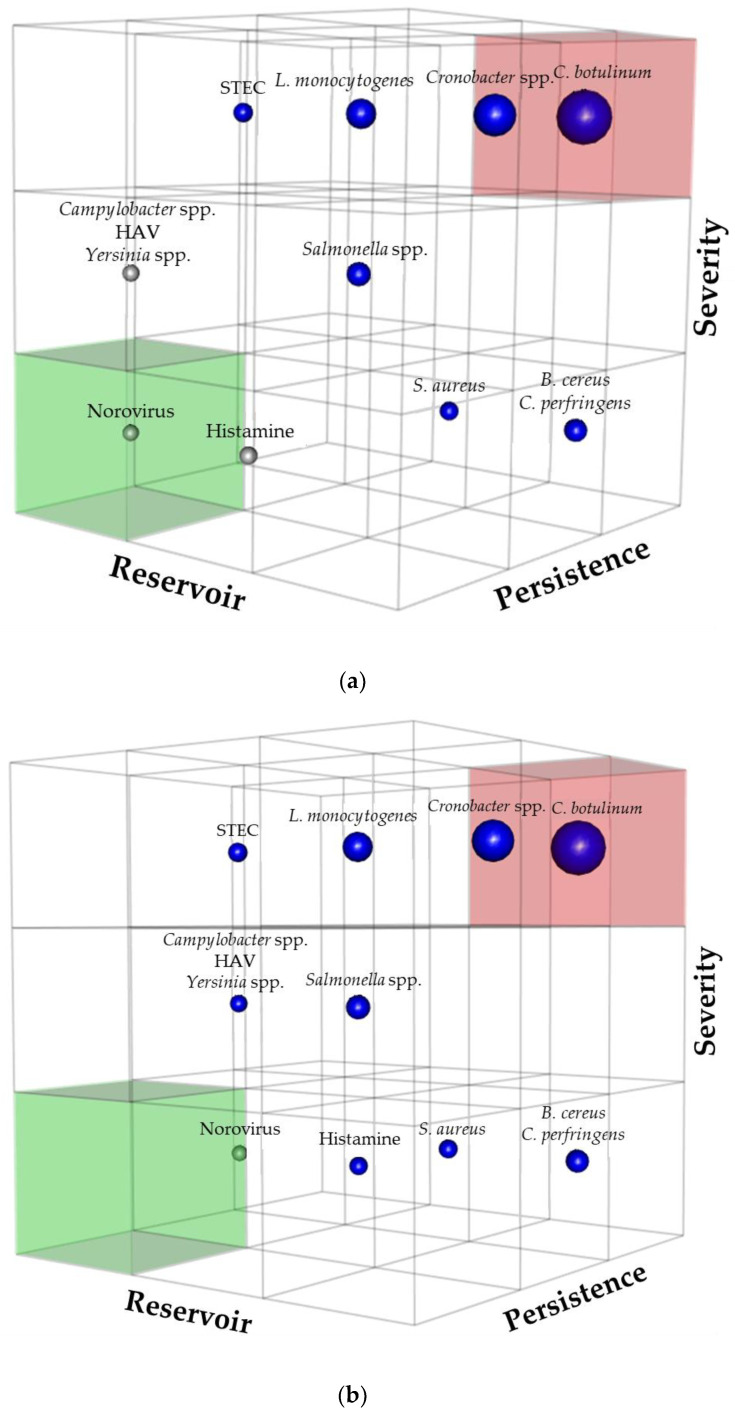
Application of the three-dimension risk matrix to potential microbiological hazards in edible insects: (**a**) Powders A, B, and C, (**b**) powder D, grey spheres represents low risk levels, up to 3, while the maximum is 125.

**Figure A1:**
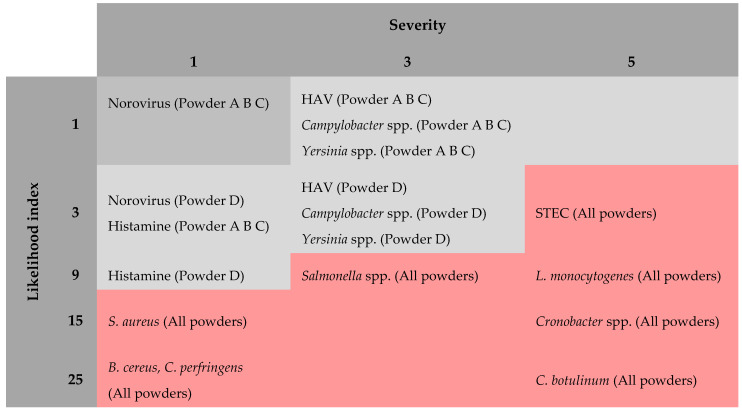
Application of the two-dimension risk matrix to potential microbiological hazards in edible insects—Hazard Analysis.

Then, we used the risk score in the classic two-dimensional matrix (Appendix A, Figure A1), developed in a three-dimension matrix to visualize all three criteria and the risk score (Severity, Reservoir, Persistence) in a unique representation (Figure 4), the volume of each sphere is correlated with each hazard’s risk score.

### 3.3. Selection of a “Short List” of Significant Hazards

Potential hazards were represented in a three-dimensional matrix (Figure 4). The traditional two-dimensional matrix is available in Appendix A, Figure A1. Significant hazards were, for all powders, in descending order of risk score:

- C. botulinum
- *Cronobacter* spp.
- L. monocytogenes
- *Salmonella* spp.
- *B. cereus* and *C. perfringens*
- *S. aureus* and STEC

The HACCP team has to identify and organize specific activities, in addition to Good Hygienic Practices, in order to eliminate or reduce these significant hazards to acceptable levels. These specific activities are control measures of biological hazard related to certain stages (Critical Control Point) of the processing flow chart [41]. The continuous monitoring of the control measures is planned in the next step of the HACCP method. This monitoring will provide evidence that the production process, as well as the significant biological hazards, are under control [29].

#### 3.3.1. *Bacillus cereus*

*B. cereus* is ubiquitous in nature, commonly found in soil and associated with improper food handling/storage, and improper cooling of cooked foods [28]. *B. cereus* causes two types of foodborne illness, an emetic (vomiting) intoxication due to the ingestion of a toxin (cereulide) pre-formed in the food and a diarrheal infection due to the ingestion of bacterial cells/spores which produce enterotoxins in the small intestine [42]. More recently, the European Food Safety Authority (EFSA) panel on biological hazards (BIOHAZ) [43] reported that most cases of foodborne diseases have been associated with greater than 10^5^ CFU/g of *B. cereus* in the food vehicle, with some cases ranging to 10^8^ CFU/g for diarrheal infection.

*Bacillus* spp. were frequently detected in various edible insects and insect-based products [32]. Fasolato et al. [44] conducted a study that was specifically focused on the identification of *B. cereus* bacteria isolated from processed edible insects (mealworms, crickets, mole crickets, and silkworms). They found that aerobic spore-forming bacterial counts were considerably high (1.6–8.1 log CFU/g with 25% of the samples analyzed), with *B. cereus* counts of 4–6.6 log CFU/g, while another study reported *B. cereus* counts >5 logs CFU/g in marketed cricket powder [45]. Furthermore, *B. cereus* spores can survive in mealworm powders and can proliferate after rehydration. Due to its abundance in soils and insects, and its resistance to industrial treatments and other stress, *B. cereus* is a major concern in the consumption of edible insects [44].

#### 3.3.2. *Clostridium botulinum*

*Clostridium botulinum* toxins are one of the most lethal substances known, which block nerve functions and can lead to respiratory failure and muscular paralysis.

*C. botulinum* spores are heat-resistant, and exist widely in the environment, and in the absence of oxygen, they germinate, grow, and then excrete neurotoxins. *C. Botulinum* toxins are thermolabiles. Foodborne botulism, caused by the consumption of improperly processed food (e.g., foods in low-oxygen-packaging or home-canned), is a rare but potentially fatal disease if not diagnosed rapidly and treated with antitoxin [46]. In addition, the bacterium can colonize and grow in the intestinal tract of some newborn infants who have not developed a desirable competing microflora, described as infant botulism [47].

Properly dried insects will not support the growth and the production of toxins of *C. botulinum* but can be a source of spores when used as ingredients. As far as we know, detection of *C. botulinum* in edible insects has not been reported, although *Clostridium* spp. have been reported in fresh *T. molitor* [48,49], processed and fresh crickets [50,51], and grasshoppers *R. differens* [52].

Nevertheless, insects are known to be a vector of *C. botulinum* spores or toxins [53,54] and fatal cases of botulism have been linked to the consumption of insects in Kenya [55].

#### 3.3.3. *Clostridium perfringens*

*C. perfringens* is a ubiquitous spore-forming bacterium distributed in nature and has been isolated from environmental sources such as water, soil, sewage, and dust. Humans and animals can carry it in their digestive tract so that foods of animal origin (such as raw meat, beef, poultry, gravies, etc.) are another frequent source [56]. *C. perfringens* produces and secretes numerous toxins and hydrolytic enzymes, including the enterotoxin responsible for food poisoning, which unlike the other toxins of this bacteria, is only synthesized during sporulation.

The dose-response is currently unknown. However, the presumptive ingestion of ≥10^8^ viable vegetative *C. perfringens* cells is sufficient to cause illness, implying that the cells survive the acidic conditions of the stomach and subsequently form spores in the large intestine and produce enterotoxins [57]. *C. perfringens* grows rapidly in a temperature range between 30 and 50 °C, and maintenance of culinary preparations for several hours in this temperature range makes possible a proliferation above 10^5^.

Although, at low levels (below 2 log CFU/g counts) *C. perfringens* spores have been reported in marketed whole processed (boiled and dried) crickets, grasshoppers, mealworms, and cricket powder [51], as well as in tenebrionid beetles [58]. As such, it is essential to pay attention to the production and conservation conditions of processed insects to suppress the presence of *C. perfringens*. Additionally, rehydration or use of *C. perfringens*-contaminated mealworm powders in other food preparations (e.g., baby porridge) is a potentially risky practice [28].

#### 3.3.4. *Cronobacter* spp.

*Cronobacter* spp. is a pathogen causing bacteremia, meningitis, and necrotizing enterocolitis associated with a high fatality rate (40~80%) in neonates via the consumption of infant formula [49]. *Cronobacter* spp. have been isolated from many foods of plant or animal origin, whether smoked, frozen, fermented, raw, or cooked, especially surviving in very dry foods (e.g., powdered infant formula, herbs, nuts, and spices). A study has shown that insect powders, which have a consistency and physicochemical properties similar to infant formula, have the same potential to be contaminated. The authors also underlined that they should be evaluated thoroughly for the presence of *Cronobacter* spp., especially when it is used for enriching the nutritional quality of children’s porridge [28]. Meanwhile, *Cronobacter* spp. was identified as a dominant Operational Taxonomic Unit (OTU) in fresh edible mealworm larvae from diverse industrial rearing companies and production cycles through a metagenetic analysis [59,60].

#### 3.3.5. *Listeria monocytogenes*

Listeriosis is one of the most severe foodborne diseases, caused by bacteria *Listeria monocytogenes*. The incidence of listeriosis is low but the high hospitalization and mortality rates (10–30%) associated with this infection make it a significant public health concern [61].

*L. monocytogenes* is a ubiquitous soil bacterium, very widespread and resistant in the environment. It is psychrotrophic and can slowly grow at refrigeration temperatures and has the ability to persist in food-processing areas and equipment.

To our knowledge, viable *L. monocytogenes* has not been isolated from edible insects by cultural methods. However, *Listeria* sp. have been detected, at relatively low abundance, in cricket powder and processed mealworm larvae from the Netherlands and Belgium [51,60], whereas *Listeria ivanovii* was found in dried *T. molitor* larvae [62]. Furthermore, *Listeria* sp. have been isolated via the Most Probable Number (MPN) method from a salted mealworm sample with a low level of contamination (~0.9 MPN/g).

#### 3.3.6. *Salmonella* spp.

*Salmonella* spp. is a major cause of foodborne illness worldwide, accounting for about 30% of foodborne outbreaks in Europe in 2018 [63]. *Salmonella* spp. reside primarily in the gastrointestinal tract of animals, including pigs, cattle, and domestic poultry, and can survive several months in fecal matter of animals and environment [64]. Transmission to humans mostly occurs through the consumption of raw or undercooked contaminated foods. Most cases of salmonellosis are mild, though it can be life-threatening, and the severity of the disease depends on host factors and the serotype of *Salmonella* spp. [65].

Several studies have shown *Salmonella* spp. absence in 25 g samples of fresh and processed edible insects [44,51,66]. Notwithstanding, this bacteria is still a major concern since Wynants et al. [67] have shown that *Salmonella* spp. can survive in the substrate used during the rearing of mealworms and can be further transmitted to the larvae. Furthermore, *Salmonella* spp. was diffusely detected among tenebrionid beetles, beetles, flies, cockroaches, crambid butterflies, house flies [58], and *T. molitor* larvae [45]. It has also been reported that Ali et al. [68] isolated *Salmonella* from fresh and fried grasshoppers in the North of Cameroon.

#### 3.3.7. Shiga-Toxin-Producing *E. coli* (STEC)

*Escherichia coli* (*E. coli*) is a Gram-negative, commonly found in the lower intestine of warm-blooded organisms [69]. Some strains of *E. coli* such as Shiga-toxin-producing *E. coli* (STEC) can cause severe foodborne disease, especially in children. STEC infections have been associated with a wide range of clinical outcomes, from mild intestinal discomfort to hemolytic uremic syndrome (HUS) or end-stage renal disease and death.

Primary sources of STEC outbreaks are raw or undercooked ground beef products, dairy products, and vegetables.

Insects are not a primary reservoir of STEC. In some studies, *E. coli* was not detected in all the tested samples, both processed and fresh insect samples. However, Kobayashi et al. has shown that *Escherichia coli* O157:H7 proliferated in houseflies for at least 3 days after ingestion, suggesting a potential dissemination mechanism [70]. Besides, during the production of insects, rearing water and ruminant-based feed ingredient supplies could be the vehicles of transmission for *E. coli*.

#### 3.3.8. *Staphylococcus aureus*

*S. aureus* is a common opportunistic human pathogen. It is widespread in nature and is a part of the saprophyte flora of the skin and mucosae of humans and animals [32]. Therefore, *S. aureus* could be present in edible insects due to contamination during handling or processing. *S. aureus* has the ability to produce numerous toxins, including staphylococcal enterotoxins (SEs, proteins only produced by those carrying certain genes), which are responsible for foodborne outbreaks associated with this bacterium [71]. Prolific growth of the bacterium is possible in the 5~40 °C range [47]. *Staphylococcus* enterotoxins (SEs) are resistant to heat-treatment, freezing, and drying.

*S. aureus* has been detected in several fresh and processed edible insect species [32], including *T. molitor* larvae [48,60,72]. *S. aureus* is sensitive to heat treatment, but processed (i.e., boiled and salted) edible insects are favorable for the growth of this species, as it is halophilic and has the ability to dominate in the absence of competition [28]. Physicochemical properties of mealworm powders are compatible with the survival or growth of *S. aureus*, since it is resistant to low water activity [34]. Given its abundance in the microflora of numerous edible insects and insect-based foods and the ability to produce heat-resistant enterotoxins, it is necessary to take some control measures to prevent the contamination and growth of *S. aureus*.

#### 3.3.9. List of Reasonably Expected Biological Hazards Excluded from the Short List and Reasons for Their Avoidance

Infected humans are the main reservoir of HAV and norovirus that are transmitted by the fecal–oral route (person-to-person or indirect). The contamination of edible insects could be prevented with the application of Good Hygienic Practices. In addition, there are no reasons to believe that these microorganisms can resist the production process of *Tenebrio molitor* powder.

*Campylobacter* spp. and *Yersinia enterocolitica* are mainly related to animal reservoirs, birds, and *Suidea*, respectively. The severity of these bacteria is similar and their resistance to environmental conditions, including insect production conditions, is not very high. Compliance with the ban on the use of pig or poultry droppings as rearing substrates combined with Good Hygienic Practices will allow the control of these hazards. Despite this, recently, Frohling et al. demonstrate the presumptive presence of *Campylobacter* spp. on untreated samples and before heat treatment of crickets (*A. domesticus*) [73]. This result confirms *Campylobacter* spp. as a potential hazard in edible insects.

Histamine formation requires a high content of free L-histidine, the presence of large quantities of histaminogenic microorganisms, and favorable physicochemical conditions (pH, water activity, temperature). It is known that insects (including *Tenebrio molitor*) are rich in histidine. According to Rumpold and Schulter [74], *Tenebrio molitor* larvae have a proportion of 35 mg of histidine per g of protein, while the tuna meat has a slightly higher proportion (5 g per 100 g of protein). It is not very clear if the natural microflora of insects contains histaminogenic bacteria. Nevertheless, the production of histamine during rearing or production of *Tenebrio molitor* powder is unlikely, due to the microbiological effect of the transformation process, combined with Good Hygienic Practices. Mealworm powder is a product intended to be stored for a long time at room temperature, due in particular to its low a_w_.

## 4. Where and How to Control Significant Hazard? A Risk-Based Approach

Managing the safety of commercialized *Tenebrio molitor* powders requires the implementation of control measures applied to significant hazards at targeted steps along the farm-to-fork chain. These control measures are used “to prevent or eliminate a food safety hazard or to reduce it to an acceptable level” at targeted steps, called Critical Control Points (CCPs) [15]. Determining CCPs corresponds to task 7 (principle 2) of the HACCP method, enabling the identification of steps where controls are critical to ensure product safety, and is followed by task 8 (principle 3) that aims to establish critical limits of these CCPs [41].

It is now recommended that the implementation of the HACCP system be undertaken in a risk-based perspective [75,76] by linking it to Food Safety Objectives (FSOs), derived from risk assessments that set appropriate levels of protection (ALOP) [77,78]. An ALOP is “the level of consumer food safety that would then be adopted as food safety policy by the national government” [78]. This criterion is linked to the Food Safety Objective (FSO) that corresponds to the maximum frequency or concentration of the hazard in a food at the time of consumption. The latter can be seen as a threshold that must not be exceeded, linked to initial levels found in raw materials (H_0_) as well as reductions (∑*R*) and increases (∑*I*) that can occur all along the farm-to-fork chain, with Equation (1) [79]:

H_0_ − (∑*R*) + (∑*I*) ≤ FSO (1)

Defining a FSO is under the responsibility of the governments of each country, as it requires the determination of an acceptable level of safety for each pair of microorganism and foods [80]. However, food business operators have the responsibility to market foods that are not harmful to consumers so they often target very low levels of FSO [80]. A strategy to progress on this objective is to use the present FSO concept and its associated criteria in conjunction with the implementation of the HACCP system in industry, by developing control measures that will maximize reductions (∑*R*) while minimizing increases (∑*I*) and initial levels found in raw materials, H_0_. This can be translated in the setting of operational Performance Objectives (PO) of food businesses’ operators that would correspond to the frequency or concentration of the hazard not to be exceeded at a specific point in the food chain. This will be monitored by the surveillance of Performance Criteria (PC) and Product Criteria (PrC). In this way, critical limits set in the HACCP system will be based on quantitative estimates as much as possible. They can be based on predictive microbiology models that will underpin expected inactivation efficiency with associated PC as well as growth limits and make it possible to validate, or demonstrate inadequate effectiveness, of specific control measures [81,82].

Tasks 7 and 8 of the HACCP method will be implemented for the present case study in a risk-based approach by first investigating levels of biological hazards in raw materials (H_0_), followed by the determination of CCP and the calculation of microbial inactivation performance of each step. Then, bacterial growth will be estimated for a selection of potential powder uses. By combining this information, the efficiency, or limitations, of specific control measures will be evaluated for the four selected insect manufacturing processes combined with the four different powder uses.

### 4.1. Initial Levels of Hazards in Raw *Tenebrio molitor*

The use of raw materials of good microbiological quality is a prerequisite to guarantee the safety of the end product. To date, data on the prevalence and level of contamination of bacteria in raw larvae are few and do not enable a proper estimation of H_0_. Main data came from larvae raised in Belgium [48,50,60,83,84,85], Italy [66,86,87], Germany [88], and the Netherlands [9,50,51,89].

Results of these analyses of unprocessed *Tenebrio molitor* larvae have shown a high level of microbial loads for the following specific indicators:

- Total mesophilic aerobes: 6.4 to 9.3 log CFU/g
- Lactic acid bacteria: 4.9 to 8.3 log CFU/g
- *Enterobacteriaceae*: 5.0 to 7.7 log CFU/g
- Bacterial endospores: <1 to 5.3 log CFU/g
- Psychrotrophic aerobic count: 5.9 to 7.6 log CFU/g
- Yeasts and moulds: 2.6 to 6.5 log CFU/g

These results were expected as *Tenebrio molitor* larvae also contain the gut. However, there is no concrete data yet on the prevalence and level of contamination of the 8 significant hazards of concern (*C. botulinum*, *Cronobacter* spp., *L. monocytogenes*, *Salmonella* spp., *C. perfringens*, *B. cereus*, *S. aureus*, and STEC). Indeed, *L. monocytogenes*, *Salmonella* spp., and *Cronobacter* spp. were not isolated from samples analyzed, while low levels of sulfite-reducing anaerobes were found, indicating potential *C. perfringens.* Though, recent studies have demonstrated the presence of *Staphylococcus* spp., *Listeria* sp. [51], and the ability of *Listeria monocytogenes* to survive and grow in this media [90,91], as well as *Salmonella* spp. [67]. Also, significant concerns regarding the *Bacillus cereus* were raised [44]. Consequently, until the microbial profile of the larvae is fully characterized, it is essential to ensure that the chain of transformation and distribution of these larvae will aim to inactivate them and limit their survival and growth.

### 4.2. Determination of CCP and Estimation of Their Related Inactivation Performance

Critical Control Points are steps “at which control can be applied and is essential to prevent or eliminate a food safety hazard or reduce it to an acceptable level” [92]. In our case study, considering the evolution of the method [93], we classified as CCP only steps that can be controlled systematically with specific critical limits. We also identified Prerequisite program(s) (PRP(s)) that are “preventive practices and conditions needed prior to and during the implementation of HACCP and which are essential for food safety” [93]. Steps that are CCP or PRP were identified, using the decision tree suggested by the European Commission [93], answering the following questions (Q) for the four investigated processes:

- Q1—Do preventive measure(s) exist?
- Q2—Is this step designed to eliminate the hazard or to reduce its occurrence to an acceptable level?
- Q3—Could contamination occur at this step or can the hazard increase to an unacceptable level, or has it occurred or increased in earlier steps and there are no earlier CCPs or PRPs?
- Q4—Can one further step eliminate the hazard or reduce its occurrence to an acceptable level?

Answers to each question are reported in Appendix A, Table A1. It has to be noted that “acceptable and unacceptable levels” mentioned in Q2 need to be defined within the overall objectives in identifying the CCPs and PRPs of the HACCP plan.

As a result, for processes A, B, and C, the step 4a of hot slaughtering was considered as a CCP as well as hot drying, step 6a, for process A and B, and cooking, step 5c, for Process B. For all processes, step 1 of reception/fasting/sieving and step 8 of packaging were considered as PRPs. First, the quality of raw materials will be crucial to ensure the safety of all products, especially process D that has no CCP, and then fasting and sieving conditions must be controlled to avoid increase. Lastly, packaging is also a PRP that needs to be carefully conducted as materials and sealing quality can influence the exchange of moisture with the environment and consequently, powder water activity and potential growth. Process D is particularly sensitive because it has no control measure that will enable to decrease the level of microorganisms present in raw materials. Consequently, it would be necessary to modify the process to include a CCP, like boiling for instance, as well as to control the quality of the raw materials and the implementation of Good Hygiene Practices at every step.

### 4.3. Inactivation Performance Achieved by Each Process Step

Hot slaughtering is applied to products A, B, and C, while cooking is applied to product B. They can both be considered as killing steps but will produce different levels of performance according to the temperature and duration applied and the characteristics of the microorganisms considered. More precisely, bacterial spores of *B. cereus*, *C. perfringens,* and *C. botulinum* could be more resistant than any vegetative cells, and the staphylococcal enterotoxin is more heat-resistant than the bacteria itself. Some spore-forming bacteria can produce toxins that are very resistant, like cereulide [94], but we do not yet have enough data to include that in our calculations. Thus, the efficiency of each inactivation step was assessed, when possible, on bacterial spore, vegetative form, and toxin, separately. The inactivation performance linked with hot slaughtering and cooking steps were assessed using the Bigelow thermal inactivation model [95] described by Equations (2)–(4):

(2) $$\mathrm{IP}=\log\frac{N_{0}}{N\left(t\right)}=\frac{t}{D_{T}}$$

(3) $$N\left(t\right)=N_{0}\;.\;10^{-\frac{t}{D_{T}}}$$

(4) $$D_{T}=D_{\mathrm{Tref}}\;.\;10^{\frac{\left(T-\mathrm{Tref}\right)}{z}}$$

where,

IP is the inactivation performance (in log CFU/g);
N_0_ is the initial level of concentration before processing (in CFU/g);
N(t) is the concentration in microorganisms at time t (in CFU/g);
t is the duration of the treatment (in hours);
D_T_ is the time of decimal reduction at temperature T (in hours);
T is the temperature of treatment (in °C).

Parameters used for each of the eight significant microorganisms are reported in Appendix A, Table A2. D-values correspond to the time to get one log reduction at a specific temperature and z-value is the rise in temperature necessary to divide by ten the time of treatment and achieve the same reduction. Spore-forming bacteria and *S. aureus* toxins are more resistant than vegetative forms as they have higher z-values and D-values.

The hot-drying step applied to processes A and B can also be considered as resulting in additional reduction, but this remains uncertain given the decrease of a_w_ during this step that increases, at the same time, the resistance of microorganisms [96]. Thus, in the risk assessment of powder infant formula, this step was, for instance, not considered as an inactivation step on *Cronobacter* spp. and *Salmonella* spp. [97]. It would be necessary to model the change in water activity in the product during the drying step and the inactivation efficiency according to the evolution of this parameter. However, this is highly variable in relation to the procedure applying to the step, as it depends on the thickness of the insect layer, the blend or not during the drying process, and the regulation system of the drying unit, all of which will influence the speed of drying and the progress in the product. This is something that, to date and to our knowledge, it is not possible to measure or estimate. Nevertheless, considering the same inactivation parameters in wet products would overestimate the inactivation, while considering parameters of dried products would underestimate the inactivation. This latter assumption corresponds to a worst-case scenario that was considered for our calculations using Equations (2)–(4), with inactivation parameters collected in dried products (Appendix A, Table A3), when available.

In addition, the software Sym’Previus (ADRIA development, France) [98] was used to compare the prediction of inactivation performance with different mathematical models. This software is a simulation tool integrating different mathematical models for predicting bacterial growth, growth/no growth boundaries, and thermal inactivation function of specific parameters, such as temperature, pH, and a_w_ of a food matrix. It provides results for different groups of *Bacillus cereus* (A, B, C, and IV), as it included detailed parameters that were not directly available in the literature, via direct calculations.

It has to be noted that models’ parameters used for direct calculations and implemented in Sym’Previus are not specific to insect’s matrices: they used data from dairy products, seafood, ready-to-eat products, meat, cooked pork meat, corn, etc. Thus, estimations must be seen as general trends and cannot be considered as precise estimates.

The inactivation performances estimated for each inactivation step are reported in Table 5. Calculations and simulations with Sym’Previus provided similar results for *C. botulinum* group I and II, *Cronobacter* spp., *E. coli*, *L. monocytogenes*, *Salmonella* spp., and vegetative form of *S. aureus*. A difference was observed for the estimate of *C. perfringens* for hot slaughtering and *C. botulinum* group II for the cooking step. That can be explained by the fact that the D-values used for calculation are higher than those implemented in Sym’Previus for *C. perfringens* and the opposite for *C. botulinum* group II. Results confirm the high resistance of spore-forming bacteria and *S. aureus* toxin as most of them achieved little reduction (<1 log CFU/g), while vegetative bacteria are well inactivated by hot slaughtering, cooking, and hot drying (for those it was possible to estimate). Regarding *B. cereus* and *C. botulinum*, different efficiencies of inactivation were found, and this highlighted the necessity of investigating at the strain level. Indeed, *B. cereus* group B is less inactivated than group C during hot slaughtering. Similarly, non-proteolytic *C. botulinum* (groups II and III) are well inactivated by hot slaughtering, while it is inefficient for proteolytic (groups I and IV).

Considering that some processes combine different inactivation steps, performances were summed per process to show the overall effect in Table 6. Process D did not provide reductions, as it does not involve an inactivation step. Processes A, B, and C provide similar inactivation performances. This is highlighted due to the fact that all three use hot slaughtering, that provides most of the reductions (Table 5). However, the same conclusions can be drawn for processes A, B, and C as for the hot slaughtering step, i.e., these processes are insufficient to inactivate spore forming bacteria and toxins if present in insects. In fact, to achieve 12 log reduction (a commonly used Sterility Assurance Level (SAL) in the food industry), this would require increasing the temperature to 130 °C for 5 min or boiling for more than one day, but both would be insufficient for *S. aureus* toxin. Considering that both options are hardly applicable except in sterilization, boiling for 5 min can be considered as efficient for vegetative bacteria and must be supported by careful monitoring of bacterial endospores. This critical limit of 5 min at 100 °C must correspond to the effective treatment applied, meaning that the 5 min does not include the time required to reach 100 °C at the core of the product.

### 4.4. Estimate of Bacteria Growth for Four Potential Powder Uses

Manufacturers are in charge of anticipating the potential uses and misuses of commercialized products (according to the law). To date, powders can be used in various recipes, as recommendations listed on the packages are wide-ranging, as are the different recipes that can be found online. These uses mainly concern baked foods, including cookies, biscuits, bread, and pastry, beverages with smoothies and drinks with high protein content, dishes with burgers or in addition to eggs and vegetables as well as chips, and pasta. It was also found to be used in baby porridge [28]. Four potential uses were investigated as a model to consider different options that will affect levels of microorganisms and different populations of concern (Figure 2).

Growth was considered negligible during the process as Good Hygienic Practices were assumed and because the low a_w_ of the product does not allow the multiplication of microorganisms. Although, in powder infant formulae, it has been found that this storage could lead to a slight reduction in the microbial load of *Cronobacter* spp. and *Salmonella* spp. [97], this was not taken into account for mealworm powders. In addition, food preparation consists in mixing ingredients that must be undertaken very quickly. Thus, only the growth after product preparation was considered in the case study calculations.

Preparation of the food items using mealworm powder, water, and additional ingredients will influence the water activity that will increase above 0.94 when mixing ingredients. This corresponds to the minimum growth parameters (Appendix A, Table A4) of significant microorganisms. Baby porridge, protein shake, and burger will maintain this high a_w_, while it will decrease for biscuits during heating to around 0.3. We must therefore consider that growth is reasonably foreseeable during the storage of prepared baby porridge, protein shake, and burger.

Growth of microorganisms can be estimated by a tri-linear model which consists of three phases: a lag phase without development, corresponding to the time of adaptation of the microorganisms to the change of environment, a second phase of exponential growth, and a third phase that is stationary when maximum density is assumed to be reached around 9 log CFU/g. The evolution of the microorganisms can be represented by the following equations [99]:

(5) $$\ln\left(N\left(t\right)\right)=\left\{ \begin{array}{ll} \ln\left(N_{0}\right) & ,t\leq t_{\mathrm{lag}}\\ \ln\left(N_{0}\right)+\mu \times (t-t_{\mathrm{lag}}) & ,t_{\mathrm{lag}}<t<t_{\max}\\ \ln\left(N_{\max}\right) & ,t\geq t_{\max} \end{array}\right.$$

where,

N(t) is the concentration in microorganisms at time t (in CFU/g);
t is the duration of storage (in h);
N_0_ is the initial level of concentration at storage (after processing) (in CFU/g);
N_max_ is the maximum concentration of microorganisms (in CFU/g);
lag is the duration of the latency phase (in hours);
µ_max_ is the maximum growth rate (in h^−1^).

Calculations reported in the present case study used simulations from Sym’Previus software that implemented the logistic model with delay and breakdown [100] to get a more adjusted prediction (Equation (6)), using the same parameters:

(6) $$\ln\left(N\left(t\right)\right)=\left\{ \begin{array}{ll} \ln\left(N_{0}\right) & ,t\leq t_{\mathrm{lag}}\\ \ln\left(N_{\max}\right)-\ln(1+\frac{N_{\max}}{N_{0}}-1)\times e^{{-\mu }_{\max}\left(t-\mathrm{lag}\right)}) & ,t>t_{\mathrm{lag}} \end{array}\right.$$

The lag time is correlated to the growth rate for a given food matrix and is specific to the strain considered [101]. The growth rate µ_max_ depends on the food temperature, pH, and a_w_, and can be estimated using a cardinal secondary model [102], Equation (7). It is equal to zero when T and/or pH and/or a_w_ are below their minimum or above their maximum values. More information to calculate gamma and interaction factors are available in References [103,104].

µ_max_ = µ_opt_ × γ(T) × γ(pH) × γ(a_w_) × ξ(T, pH, a_w_) (7)

where,

µ_max_ is the maximum growth rate in current conditions (in h^−1^);
µ_opt_ is the growth rate at optimum conditions (in h^−1^);
γ(T), γ(pH), and γ(a_w_) are cardinal values ranging from 0 to 1, equals 1 at optimum conditions;
ξ(T, pH, a_w_) is an interaction factor also ranging from 0 to 1.

Growth was estimated for an initial level of contamination of 0 log CFU/g, meaning 1 CFU/g. The results of each potential use are reported in Table 7. While T, pH, and a_w_ parameters are specific to the microorganism, the µ_opt_ is specific to the microorganism/matrix pair. As for inactivation, the results should be interpreted as general trends as model parameters implemented in Sym’Previus were collected in dairy products, seafood, ready-to-eat products, meat, cooked pork meat, and corn.

The estimates show that many of the simulated scenarios allow the growth of the eight microorganisms of interest, with the exception of biscuits, which have a low a_w_ (<0.3).

Baby porridge shows growth of less than 0.7 CFU/g when consumed within one hour maximum and left at an ambient temperature of 20 to 30 °C. It can reach almost 3 log CFU/g if it is left for up to 4 h. It is therefore essential to consume it quickly, within one hour following preparation.

Protein shake is also intended to be stored at room temperature, often for longer periods when it accompanies sports sessions or when it is consumed throughout the day. Thus, high growth levels are observed for all microorganisms at 30 °C, ranging from 1.1 log CFU/g for 3 h of storage to 5.3 log CFU/g for 8 h of storage. These values are of high concern and justify storage recommendations of less than 3 h as well as enhanced monitoring of microorganisms that may be present in the mealworm powder.

The storage of prepared burgers shows limitations in particular at 8 °C, *Listeria monocytogenes* reaches 2.5 log CFU/g in three days and *C. botulimum* type II, 3.8 log CFU/g. Other microorganisms show no growth at 4 °C and little or no growth at 8 °C (<1 CFU/g). However, *Listeria monocytogenes* is not expected to be present if the powder has been heat-treated, as suggested by inactivation calculations.

### 4.5. Safety of Powders A, B, C, and D

Combining findings from the analysis of the initial contamination of *Tenebrio molitor*, CCPs determination, calculations of inactivation performance of each process, and growth estimates of four different potential uses, provides essential information substantiating the safety of insect powders. Initial raw material contamination data show high levels of contamination. This justifies the use of a process including CCPs of inactivation. Thus, process D using cold slaughter and freeze-drying must be performed in a very precautionary manner in order to produce safe products with, in particular, a very strict application of good hygiene practices and a cold chain storage with a short shelf life and could give rise to stricter safety criteria. Processes A, B, and C benefit from the hot slaughter that allows inactivation of vegetative bacteria. This step must be monitored very carefully to ensure that the critical limits of 5 min at 100 °C are met. However, this does not reduce spore-forming bacteria including *B. cereus*, *C. botulinum,* and *C. perfringens,* while a consideration of different possible uses has shown that they can multiply during the storage of prepared products. It is therefore essential to consider the possible uses of the powder and to strengthen the inactivation step, for instance by sterilization. It is also necessary to strengthen controls on spore-forming bacteria in raw materials and to continue research to characterize the quality of raw materials.

## 5. How to Be Sure It Is Working All the Time? Monitoring System

According to Codex Alimentarius, task 9 of the HACCP method is to “Establish a monitoring system for each CCP”. This is a formalized set of observations and/or measures for the monitoring of each step of the flow diagram considered at CCP and relative to critical limits (task 8). Monitoring methods, materials, and procedures must be selected considering the validity of the measurements and the establishment of a metrological plan. In particular, procedures for calibration, verification, and maintenance of the equipment used must be available. The chosen monitoring system can detect an uncontrolled production situation and provides assurance of product safety.

In our case study, we have 3 steps of the flow chart considered as CCPs (i.e., 4a hot slaughtering, 5b cooking, and 6a hot drying). One of them is common to powders A, B, and C: hot slaughtering. A ratio of larva:water of 1:1 is placed in a churn tank with boiling water and temperature sensors inside. Temperature is continually recorded during the 5 min of slaughter. A second similar equipment is used for the cooking step (step 5b). Temperature is also continually recorded during the 30 min of cooking. For hot drying (step 6a), the dryer used for this step will receive either a batch of defatted insect paste or a batch of whole killed insects. Both are spread in thin layers in the dryer, for an extended period of time. Two parameters can be monitored: the temperature of the insect paste using sensors or the water activity of a paste sample. The measure of a_w_ requires simple equipment (a_w_ meter) and is rapid (5 min), which is compatible with CCP monitoring. An alternative system of monitoring is the continuous checking of the water content of the air leaving the dryer.

Then, task 10 is to “establish corrective actions”. These are defined as “any action that should be taken when the result of monitoring detects an uncontrolled production situation” (loss of control of a CCP). The corrective actions must ensure that the CCP has been brought under control, and also include proper disposition of the concerned product. This task must be highly structured and organized because on the one hand, it may involve, if necessary, withdrawing non-compliant products from production and distribution (and keeping proof of this), and, on the other hand, it involves reviewing deviations at regular intervals in order to know which ones occur most often so that they can be remedied. When monitoring shows a loss of control of a CCP, the HACCP team has to assess the consequences of this lack of control, in order to identify the right, balanced corrective actions. Withdrawing products is not the only option, in certain cases, corrective actions can be applied during production under the supervision of a line operator. In our case, readjustement of the temperature and/or the time, and repeating the step are decided by the line production manager according to their expertise in edible insects production processes.

Generally, the end of this task 10 is a milestone of the HACCP plan and can be formalized through the implementation of what is commonly called the “CCP control plan” (Table 8).

Subsequently, task 11 consists in establishing a verification procedure. This verification is often defined as “the application of methods, procedures, tests, and other evaluations, in addition to monitoring, to determine compliance with the HACCP plan”. In fact, it will verify both the conformity and the effectiveness of the system put in place in a true continuous improvement process. It includes four activities:

(i) Validation of the HACCP plan,
(ii) HACCP audit systems,
(iii) Equipment calibration,
(iv) Targeted sampling and analysis.

Validation is the action that evaluates whether the HACCP plan identifies and controls significant hazards in the production for which it was established [15]. This includes verifying that valid and recent scientific data and information have been used for Steps 6 and 8; if not, a scientific study should be established to conduct the verification.

Audits are systematic reviews that include on-site observations, interviews, and an examination of records to ascertain the reality and execution of the procedures implemented. The calibration of equipment should be carried out against a reference standard if possible. The absence of such a calibration for equipment linked to a CCP may lead to it being considered as uncontrolled. Finally, targeted analyses (physico-chemical, microbiological) of raw materials or finished products are of interest when they are used as part of a HACCP plan verification.

An important part of this step 11 will involve CCPs and other important points. Their highly strategic character in the control of the safety of the food produced explains why particular care must be taken in their verification. It will therefore be necessary to carry out, under real but perfectly supervised production conditions, tests simulating the loss of control at this stage. The ability of the monitoring system to detect deviation and the knowledge, by the person in charge of monitoring, of the procedures to be followed in case of loss of control of a CCP will be the main elements to be evaluated.

Validation should be a scheduled activity with a certain frequency but given the multiplicity of factors requiring the revision of the HACCP plan (changes in raw materials, suppliers, materials, recurring deviations, customer returns, changes in regulations, new scientific data, etc.), it becomes a quasi-continuous activity.

Finally, task 12 comprises the establishment of documentation and proof. The implementation of a documentation system is important to demonstrate both the compliance and the validity of the HACCP plan in place. This can be used as evidence if necessary. Well-maintained and regularly updated records are always a sign of good organization and seriousness of the organization. There are four main types of records that must be kept as part of a HACCP system:

- Basic documentation used to draw up the HACCP plan, including the documents relating to food regulation (System documentation).
- Documentation of the methods and procedures used, including a description of the monitoring systems selected for CCPs and other points and the related corrective actions and improvement actions that have been planned (Working documents).
- All information resulting from the implementation of the HACCP system, including monitoring records of CCPs and other points, as well as related records, and verification/validation records (Dynamic documents).
- Information relating to staff training programs. Beyond the traditional “hygiene” training provided in organizations, whose contents and evaluation of knowledge must be archived, there is a need to adequately train operators involved in monitoring, corrective and improvement actions, and verification necessary for the control of CCPs and other points.

In the present case study, temperature records, the analyses of a_w_ of insect paste, the measures of the water content of the air exiting the dryer, and results of microbial analysis will serve as proof of the control of biological hazards.

## 6. Discussion

Ensuring the safety of edible insect powders of *Tenebrio molitor* larvae for human food remains particularly challenging. Indeed, insects are vectors of several microorganisms, while manufacturing processes do not allow their complete inactivation, and the wide range of potential uses can allow their multiplication. Thus, a risk-based approach considering the whole farm-to-fork chain is necessary to control human health risks. The present case study included the application of the HACCP method together with the use of predictive microbiological models. To illustrate different scenarios of interest, four processes were studied including a step of hot or cold slaughtering and a hot or cold drying step. One of these processes also included a step of cooking followed by a centrifugation enabling oil extraction, prior to drying steps. These four processes have been studied with regards to four potential uses by consumers: by infants in baby porridge, by children and adults in biscuits or burgers, and by adults in high-protein shakes.

The hazard analysis was conducted using a semi-quantitative risk-ranking approach estimating on one hand a score corresponding to the likelihood of occurrence, based on its potential presence in reservoir and persistence along the process, and on the other hand, a score of severity for human health. Combining both scores, thirteen hazards were ranked and the first eight were selected as of particular concern, to be controlled by specific activities related to important steps. The list of eight significant biological hazards comprises: *B. cereus*, *C. botulinum*, *C. perfringens*, *Cronobacter* spp., *L. monocytogenes*, *Salmonella* spp., *S. aureus*, and STEC.

It has to be noted that this list should be reconsidered depending on the specific insect process as each combination of processes and potential uses could lead to a different list of significant hazards.

All processes were then analyzed to define Critical Control Points (CCPs) steps that can eliminate or reduce the likely occurrence of significant hazards identified. Among them, the step of hot slaughtering applied in processes A, B, and C and the step of hot drying applied in processes A and B were considered as CCPs. The inactivation performance of each processing step was calculated using predictive microbiological models. It revealed that *B. cereus*, group I of *C. botulinum,* and *C. perfringens*, were particularly of concern, as they are highly resistant to heat treatment. Indeed, hot slaughtering at 100 °C during 5 min did not inactivate these spore-forming bacteria (less than 1 log CFU/g). A high variability was observed between *B. cereus* groups, with group B leading to less than 1 log CFU/g of inactivation, groups A and IV to less than 5 log CFU/g, while group C had more than 9 log CFU/g. On the other hand, vegetative forms of *Cronobacter* spp., *L. monocytogenes*, *Salmonella* spp., STEC, and *S. aureus* were easily inactivated when boiling insects even for five minutes (>12 log CFU/g). This is not the case for the toxin produced by *S. aureus* that was resistant to these treatments (<0.1 log CFU/g). Increasing slaughtering time even for more than one hour was still not sufficiently efficient (less than 5 log CFU/g of some spore-forming bacteria), while increasing the temperature seemed to be more promising. For example, a 5-min treatment at 130 °C would be sufficient to inactivate 12 log CFU/g. However, this high temperature is difficult to obtain: it requires increasing the pressure with discontinuous processes or using an autoclave with a packaged product, which represents difficulties related to the risk of asphyxiating the larvae. These treatments are not easily applied to this matrix and would require additional studies to assess the impact on the nutritional and organoleptic qualities of the product. The efficiency of the step of hot drying at 100 °C during 6 h was estimated only for *Cronobacter* spp., STEC, and *Salmonella* spp., in absence of data for others. It was considered efficient among them with more than 12 log CFU/g. Thus, processes A, B, and C seem to be efficient with regards to vegetative bacteria but not to spore-forming bacteria, requiring a special attention to the quality of raw materials but also to the performance of the heat treatment. Indeed, heat transfer between the boiling water and the insect must be taken into account to determine the start of the treatment, when the core temperature of the larvae reaches the 100 °C mark. Process D does not include any CCP, requiring a stronger surveillance of Good Hygienic Practices and better microbiological quality of raw materials, including the substrate, and the potential addition to a heat treatment step.

Consecutively, the potential growth of significant microorganisms was estimated considering four potential uses and worse-case scenarios for baby porridge, biscuit, burger, or high-protein shake. During powder storage, its low water activity did not enable bacteria growth, but cells remained alive and able to multiply once the powder was rehydrated in food preparation. This can be particularly risky in the case of consumption through protein shake and baby porridge for a few hours at ambient temperature. For these two cases, *B. cereus* can grow up to 5 log CFU/g if left for 4 h at 30 °C and even 12 log CFU/g in extreme scenarios when left for 8 h. Additionally, STEC, *Salmonella* spp., *Cronobacter* spp., *L. monocytogenes*, *C. botulinum* (proteolytic and non-proteolytic), and *C. perfringens* could also be subject to significant levels of growth, if present in the product. Consumption of these products within the hour after preparation would help to avoid these potential grows. For burger, stored at refrigerated temperature, non-prot *C. botulinum*, *C. perfringens,* and *L. monocytogenes* had potential growth, especially if the temperature is around 8 °C. It is therefore important to keep the storage temperature as low as possible. When the powder is used to produce biscuits, it is quickly cooked, leading to a decrease of water activity, thus no growth was assumed.

**Figure 5:**
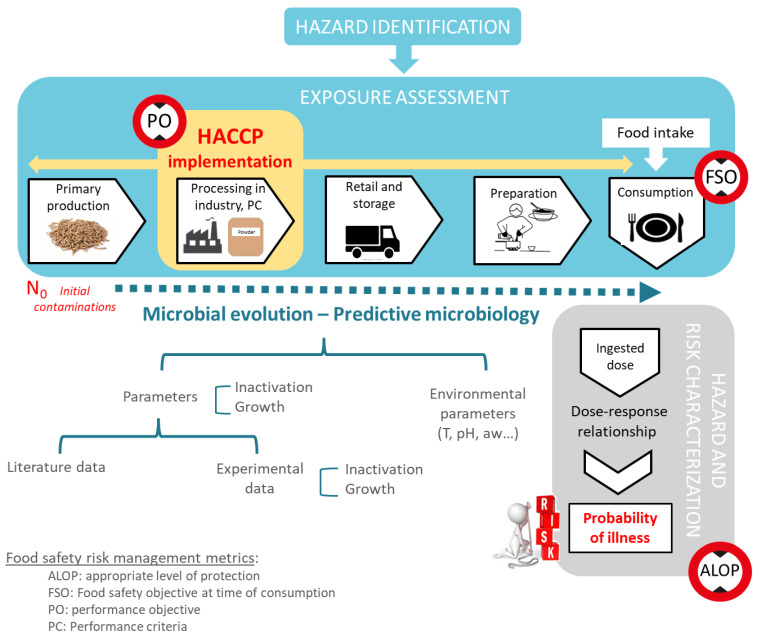
General outline of the risk-based approach implemented in the *Tenebrio molitor* HACCP case study.

Finally, the present HACCP plan was developed in a risk-based approach to control the safety of *Tenebrio molitor* for human consumption. Thus, it combined the hazard identification with the quantitative estimation of the evolution of the microbial levels along the farm-to-fork chain, using predictive models. It clearly demonstrated a higher concern regarding spore-forming bacteria than for *Salmonella* spp., although to date this is one of the most popular criteria searched in this matrix. This could be further developed using the food safety risk management metrics that are represented in Figure 5: Performance Objective (PO), Food Safety Objective (FSO), and appropriate level of sanitary protection (ALOP). An ALOP is a level of protection deemed appropriate by the country establishing a sanitary measure to protect human life or health within its territory, this concept may otherwise be referred to an “acceptable level of risk”. Hazard-based management parameters were first implemented, including control measures for operators and microbiological criteria defined by European regulations. The FSO is derived from ALOP, it corresponds to the maximum frequency or concentration of the hazard in a food at the time of consumption. For a more operational management, the PO is derived from the FSO and corresponds to a frequency or concentration of the hazard not to be exceeded earlier in the chain at a specific point. FSOs and POs are a means of communicating public health objectives (ALOP) to be achieved by food producers through the application of good practices and HACCP.

Finally, results of the present case study can be a great source for quantitative microbial risk assessment and could be now developed further including different scenarios of food intake and dose-response relationship to estimate the related probability of illness in consumers.

The main recommendations to control *Tenebrio molitor* insect powder safety for human health, for manufacturers and official control and surveillance services, are to:

- Focus on raw material microbial quality. This is clearly related to the quality of the larval-rearing substrate used, the environmental conditions of farming, and the screening and removal of dead larvae prior to transformation. Good Hygienic Practices remain a prerequisite at all these steps.
- Validate the efficiency of the process, and particularly to CCP, with regards to the eight selected hazards. This requires that cooking times as well as water:insect ratios be complied in order to optimize heat transfer. Indeed, it is important to make sure that each larva in the batch receives the established treatment, and not to start timing the duration of the treatment as soon as the larvae are put in the water, because of the time required to allow for heat transfer. These time/temperature combinations can ideally be recorded continuously by sensors and microbiological analyses of products. Furthermore, this can be combined with inactivation calculations applying to each stage in order to optimize treatment time and temperature and their efficiency. It can also be validated in industry by the use of surrogates.
- Consider powder storage in its packaging and potential use. Powders can benefit from a long shelf life, stored at ambient temperature, if the packaging remains in perfect condition, with no leaks, and is stored in good conditions, with no possibility of re-moistening. Then, considering different potential uses is a crucial point as product pH is close to neutral and water activity increases during food preparation.

On top of that, specific microbial analysis can be suggested at different stages of the process. This could include indicators like counts in total aerobic bacteria, aerobic spores, *Enterobacteria,* and sulfite-reducing anaerobic bacteria. The first point of control would be the larvae-rearing substrate as it can play a crucial role because it is consumed by insects, and some particles will obviously be introduced into the process even when insects are rinsed. Its initial quality can be assessed at reception and throughout the life cycle. It can also be done at first to compare different suppliers and then by following a regular control plan. Frequency of control and lists of indicators can be revised and spaced in time if good results are obtained. These same indicators can be used for raw material characterization at reception and time of use and to validate the efficiency of inactivation steps. The limits that will be set must take into account the fact that the grinding of the larvae will release the microorganisms in the mixture, while the drying process will concentrate them in the product with yields of only 20% to 30%, i.e., levels of contamination up to three times higher after drying than before. Then, the product shelf life must be validated and regularly controlled using these indicators as well as the research into the significant hazards depending on the expected use of the product. More broadly, cleaning procedures must be validated, in particular for the part of the process that comes after the drying of the powder because there is no more inactivation after that. The surveillance of the processing environment must be monitored to detect possible reservoirs of microorganisms that could contaminate the product. For this purpose, additional investigations are recommended. This may include searches for *Salmonella* spp., *Cronobacter* spp., and *Listeria monocytogenes*.

Furthermore, a non-exhaustive list of parameters of the manufacturing process must be checked and recorded. It includes the duration and temperature of fasting conditions and the potential implementation of a larvae-rinsing step. The CCP hot slaughtering can be monitored with measurement of duration and temperature at the core of the product and compliance with a defined ratio of water with larva. This stage also requires the setting up and control of a mixing system that will facilitate heat transfer and a more homogenous treatment. The CCP hot drying can be monitored with the water content in the dryer, duration, and temperature at the heart of larva, complying with a maximum thickness of layer and with a mixing frequency when needed (in case of high density to avoid crumbling). At the end of drying, water activity must be below 0.5. Ultimately, storage after packaging, a PRP, will be secured by the choice of the right materials and seal for the bag to avoid re-moistening, that can be controlled by checking its integrity and the quality of the weld.

This case study can be used as a model to establish a HACCP plan of biological hazards for insect powders bearing on other insect species. Also, this HACCP plan must be expanded to consider other types of hazards: allergens and chemical and physical hazards. Finally, although there is recent and promising research underway, many questions remain unanswered or would need complementary research. The main topics of research which need to be identified are:

- Identification and quantification of initial hazards and their prevalence in larvae.
- Better characterization of this matrix, specifying its pH, fat content, viscosity, and others.
- Study of the effect of applying a fasting step and method development to define different fasting conditions.
- Understand the substrate maintenance and its effect on product quality, including guidance on composition and regeneration rules.
- Study of the effect and efficiency of rinsing lavae, considering water temperature, different methods, and ratio of water:insects.
- Optimization of the heat transfer during hot slaughtering with measurement of the change in temperature at the heart of larvae for different settings.
- Estimate the efficiency of the hot drying process, especially on spore-forming bacteria, and understand the change in water activity during the drying step.
- Investigate other inactivation processes to enable spore destruction, like sterilization, and also evaluate effects on insect dough organoleptic properties.
- Collect data for predictive microbiological models of growth and inactivation in insect matrix, preferably in industry or research pilots.
- Investigate different possibilities for the commercialization of mealworm powders and resulting consumer uses to understand which foods will be substituted by this product and under which conditions they will be prepared.
- Conduct a whole biological risk assessment of insect powder consumption.

On a broader scale, consuming insect powder can also impact human health, as it can be a source of allergenic compounds, of chemical contaminants, or even to beneficial nutrients. Thus, a risk–benefit assessment would be required to estimate the overall health impact of consuming insect powder and more precisely, to estimate the change in health impact considering foods substituted by this new product. Furthermore, insect consumption is often recommended for its reduced environmental impact compared to the conventional meat product chain, and this should be assessed more specifically. This could be accomplished through a more holistic approach that would encompass health, environmental, and economic impacts at the same time.

## Figures and Tables

**Table 1:** *Tenebrio molitor* powder description.

Products	Yellow Mealworm Powder	
Raw materials	Yellow mealworm, *Tenebrio molitor* (*Coleoptera: Tenebrionidae*) fed mainly on cereal bran or flour (wheat, oats, maize) supplemented with fruits and vegetables	
Nutritional composition	Whole mealworm powder [20,21] Proteins: 48–64% Lipids: 28–36% Ashes and carbohydrates: 6–10%	Defatted mealworm powder [22,23,24] Proteins: 65–70% Lipids: 12–20% Ashes and carbohydrates: 8–12%
Product characteristics	pH range of 6.5 to 7.0 a_w_ of <0.50 and moisture content of <6% [23]	
Packaging	Packed in a hermetically sealed and opaque plastic bag	
Destination	Human consumption	
Labeling	Contains allergens similar to crustacean	
Shelf life	Best if used within 6 months from manufacturing date *	
Storage conditions	Stored in a dry, cool, and clean environment in the original unopened bags	

* Based on current shelf life of *Tenebrio molitor* powder available on the market and estimates made on powder of black soldier fly larvae *Hermetia illucens* [25].

**Table 2:** Description of manufacturing steps.

Step Name	Powder				Manufacturing Step Description
	A	B	C	D	
1—Reception mealworms	√	√	√	√	At reception, batches are visually checked, a natural yellow-brown color of the larvae indicates animals in good health, while the presence of black larvae often coupled with a strong odor reveals inadequate rearing or/and storage conditions. In the latter cases, the batch is isolated and destroyed.
2—Fasting	√	√	√	√	A 24 h fast is carried out to empty the digestive contents of insects.
3—Sieving	√	√	√	√	Sieving is performed to eliminate residues of substrates and frass. This step may also include a rinsing of larvae with water.
4a—Hot slaughtering	√	√	√		Slaughter by immersion of insects in boiling water at 100 °C for 5 min, with an insect:water ratio of 1:1, and drained.
4b—Cold slaughtering				√	Slaughter by freezing insects during 4 h at −18 °C, the thickness of the insect layer should be less than 5 cm.
5a—Mincing		√			Mincing is performed with a grinder.
5b—Cooking		√			Cooking occurs in a thermostatically controlled double-wall and agitated tank at 80 °C during 30 min, using water.
5c—Centrifugation		√			Fractions are separated by centrifugation to obtain oil and paste.
5d—Cooling			√		Boiled insects are placed for 5 min in a cold-water cooling system at 15 °C.
6a—Hot drying	√	√			Drying concerns whole insects or insect paste. The time–temperature schedule is 100 °C during 6 h. At the end, water activity must be below 0.5.
6b—Freeze drying			√	√	Freeze drying is applied to whole insects.
7—Grinding	√	√	√	√	Grinding to obtain a fine powder.
8—Packaging/Storage	√	√	√	√	Packaging in a plastic multilayer bag and storage at ambient temperature.

√ Means that the step is applied in the process of the considered powder (A, B, C or D).

**Table 3:** List of reasonably expected biological (or from biological origin) hazards, their reservoir, and general persistence (product, process) (from References [30,32,33,34]).

Hazards (Bacteria and Their Toxin, Viruses, and Metabolites)	Reservoir	Persistence in the Product and along the Process
*Bacillus cereus*	Environment (soil)	Spores resistant to heat and drying
*Campylobacter* spp.	Poultry Cattle Pigs	Heat-sensitive
*Clostridium botulinum*	Environment (Soil)	Spores resistant to heat and drying
*Clostridium perfringens*	Environment (soil) Animals’ digestive tract	Spores resistant to heat and drying
*Cronobacter* spp.	Environment (soil, dust)	Heat-sensitive Persistence in powder
HAV *	Humans	Heat-sensitive
Histamine	Produced by microorganisms in foods containing free histidine	Histamine: Heat-resistant Histaminogenic microorganisms: heat-sensitive
*Listeria monocytogenes*	Environment	Heat-sensitive
Norovirus	Human	Heat-sensitive
*Salmonella* spp.	Poultry Cattle Pigs Birds	Heat-sensitive Persistence in powder
*Staphylococcus aureus*	Skin and mucus of humans and animals Environment	Enterotoxins resistant to heat, drying, freezing
STEC **	Cattle Sheep	Heat-sensitive
*Yersinia* spp. (enteropathogenic)	Pigs Birds	Heat-sensitive

* Hepatitis A Virus, ** Shiga-Toxin-producing *Escherichia coli*.

**Table 4:** Establishment of Likelihood index (R × P) and Risk score for each potential hazard of all the powders of *Tenebrio molitor*.

Product	*Tenebrio molitor* Powders A, B, C					*Tenebrio molitor* Powder D				
Hazards	Reservoir (R)	Persistence (P)	Likelihood ^1^ (Li = RxP)	Severity ^2^ (S)	Risk ^3^ (LixS)	Reservoir (R)	Persistence (P)	Likelihood ^1^ (Li = RxP)	Severity ^2^ (S)	Risk ^3^ (LixS)
*B. cereus*	5	5	25	1	**25**	5	5	25	1	**25**
*Campylobacter* spp.	1	1	1	3	**3**	1	3	3	3	**9**
*C. botulinum*	5	5	25	5	**125**	5	5	25	5	**125**
*C. perfringens*	5	5	25	1	**25**	5	5	25	1	**25**
*Cronobacter* spp.	5	3	15	5	**75**	5	3	15	5	**75**
HAV	1	1	1	3	**3**	1	3	3	3	**9**
*Histamine*	3	1	3	1	**3**	3	3	9	1	**9**
*L. monocytogenes*	3	3	9	5	**45**	3	3	9	5	**45**
*Norovirus*	1	1	1	1	**1**	1	3	3	1	**3**
*Salmonella* spp.	3	3	9	3	**27**	3	3	9	3	**27**
*S. aureus*	3	5 *	15	1	**15**	3	5 *	15	1	**15**
STEC	1	3	3	5	**15**	1	3	3	5	**15**
*Yersinia* spp.	1	1	1	3	**3**	1	3	3	3	**9**

^1^ Likelihood index (Li = R × P); ^2^ DALY (Disability Adjusted Life Year) based; ^3^ the risk score is the product of Likelihood index and Severity score; * Score attributed considering *S. aureus enterotoxin*.

**Table 5:** Estimation of inactivation performance of each heat treatment step in log CFU/g.

Biological Hazards	Hot Slaughtering 100 °C, 5 min		Cooking 80 °C, 30 min		Hot Drying 100 °C, 6 h
	Calcul	Sym’Previus	Calcul	Sym’Previus	Calcul
*B. cereus*	6.3		1		
A		4.9		0.1	
B		0.8		0.01	
C		10.1		0.6	
IV		3.7		0.04	
*C. botulinum*			0.01		
I	0.03	0.2	0.002	0.01	
II	>12	>12	>12	0.8	
III	>12		1.2		
IV	1.8		0.1		
*C. perfringens*	0.1	2.4	0.02	0.2	
*Cronobacter* spp.	>12	>12	>12	>12	>12
*E. coli* *	>12	>12	>12	>12	>12
*L. monocytogenes*	>12	>12	>12	>12	
*Salmonella* spp.	>12	>12	>12	>12	>12
*S. aureus*	>12	>12	>12	>12	
*S. aureus* (toxin)	0.03		0.05		

* Simulations and calculations were performed for *E. coli* without specific data on STEC.

**Table 6:** Estimation of inactivation performance of the whole process of each product in log CFU/g.

Biological Hazards	Process A		Process B		Process C		Process D	
	Calcul	Sym’Previus	Calcul	Sym’Previus	Calcul	Sym’Previus	Calcul	Sym’Previus
*B. cereus* (spores)	6.3		7.3		6.3		0	
A		4.9		5		4.9		0
B		0.8		0.8		0.8		0
C		10.1		10.7		10.1		0
IV		3.7		3.7		3.7		0
*C. botulinum* (spores)								
I	0.03	0.2	0.03	0.2		0.03		0
II	>12	>12	>12	>12		>12		0
III	>12		>12			>12		
IV	1.8		1.8			1.8		
*C. perfringens*	0.1	2.4	0.1	2.4	0.1	2.4	0	0
*Cronobacter* spp.	>12	>12	>12	>12	>12	>12	0	0
*E. coli* *	>12	>12	>12	>12	>12	>12	0	0
*L. monocytogenes*	>12	>12	>12	>12	>12	>12	0	0
*Salmonella* spp.	>12	>12	>12	>12	>12	>12	0	0
*S. aureus*	>12	>12	>12	>12	>12	>12	0	0
*S. aureus* (toxin)	0.03		0.08		0.03		0	

* Simulations and calculations were performed for *E. coli* without specific data on STEC.

**Table 7:** Estimation of relative bacteria growth for each product use, with N_0_ = 1 log CFU/g (additional log CFU/g).

Biological Hazards	Baby Porridge		Protein Shake		Burger		Biscuits
	pH 7, a_w_ 0.99		pH 7, a_w_ 0.99		pH 7, a_w_ 0.99		a_w_ 0.3
	1 h (30 min–4 h)		6 h (3–8 h)		2 days (1–3 days)		3 days
	20 °C	30 °C	20 °C	30 °C	4 °C	8 °C	20 °C
*B. cereus*	0.2 (0.1–1) *	0.6 (0.3–2.5)	1.5 (0.7–2.0)	3.7 (1.9–5.0)	0 (0–0)	0.7 (0.3–1.0)	0
*C. botulinum (type I)*	0.2 (0.1–0.6)	0.4 (0.2–1.7)	0.9 (0.5–1.3)	2.5 (1.2–3.3)	0 (0–0)	0 (0–0)	0
*C. botulinum (type II)*	0.4 (0.2–1.6)	0.6 (0.3–2.2)	2.4 (1.2–3.2)	3.3 (1.7–4.5)	0.1 (0–0.1)	2.5 (1.3–3.8)	0
*C. perfringens*	0.1 (0.1–0.5)	0.6 (0.3–2.6)	0.8 (0.4–1.0)	3.8 (1.9–5.1)	0 (0–0)	0 (0–0)	0
*Cronobacter* spp.	0.2 (0.1–0.9)	0.7 (0.3–2.6)	1.3 (0.7–1.7)	4.0 (2.0–5.3)	0 (0–0)	0 (0–0)	0
*E. coli*	0.2 (0.1–0.7)	0.5 (0.2–1.9)	1.1 (0.5–1.5)	2.9 (1.5–3.9)	0 (0–0)	0.4 (0.2–0.5)	0
*L. monocytogenes*	0.2 (0.1–0.7)	0.4 (0.2–1.5)	1.1 (0.6–1.5)	2.3 (1.1–3.0)	0.5 (0.3–0.5)	1.7 (0.8–2.5)	0
*Salmonella* spp.	0.2 (0.1–0.9)	0.6 (0.3–2.2)	1.2 (0.7–1.7)	3.3 (1.7–4.4)	0 (0–0)	0.6 (0.3–0.9)	0
*S. aureus*	0.2 (0.1–0.7)	0.5 (0.2–1.9)	1.1 (0.6–1.5)	2.8 (1.4–3.8)	0 (0–0)	0.5 (0.2–0.7)	0

* As an illustration, the result “0.2 (0.1–1)” means that a storage at 20 °C during 1 h led to an increase of 0.2 log CFU/g, 30 min led to 0.1 log CFU/g, and 4 h to 1 log CFU/g.

**Table 8:** Example of HACCP control plan for the case study of *Tenebrio molitor* powders.

CCP *	Critical Control Measure	Critical Limit	Monitoring System	Corrective Actions
4a. Hot slaughtering	Thermal treatment	Water temperature 100 °C Time 5 min	Digital time/ temperature data logger	Readjust temperature or time Batch destruction
5b. Cooking	Cooking (thermal treatment)	Temperature 80 °C Time 30 min	Digital time/ temperature data logger	Readjust temperature or time Batch destruction
6a. Hot drying	Duration of hot drying. Thermal treatment. AND a_w_ on end product	Temperature 100 °C Minimum drying time of 6 h a_w_ < 0.5	Digital time/ temperature data logger Measure of a_w_	Readjust temperature or time Restart the step Batch destruction

* Critical Control Point.

## Appendix A

**Table A1:** Identification of CCPs for each *Tenebrio molitor* powder manufacturing process.

	Manufacturing Step	Q1	Q2	Q3	Q4	CCP
**PROCESS A**	1-2-3 Reception/Fasting/Sieving	Yes	No	Yes	No *	PrP
	4a Hot slaughtering	Yes	Yes (stop)	-	-	Yes CCP
	6a Hot drying	Yes	Yes (stop)	-	-	Yes CCP
	7 Grinding	Yes	No	No (stop)	-	No
	8 Packaging	Yes	No	Yes	No *	PrP
		Yes	No	Yes	No *	PrP
**PROCESS B**	1-2-3 Reception/Fasting/Sieving	Yes	No	No (stop)	-	No
	4a Hot slaughtering	Yes	Yes (stop)	-	-	Yes CCP
	5a-5b-5c Mincing + Cooking + Centrifugation	Yes	Yes (stop)	-	-	Yes CCP
	6a Hot drying	Yes	Yes (stop)	-	-	Yes CCP
	7 Grinding	Yes	No	No (stop)	-	No
	8 Packaging	Yes	No	Yes	No *	PrP
**PROCESS C**	1-2-3 Reception/Fasting/Sieving	Yes	No	Yes	No *	PrP
	4a Hot slaughtering	Yes	Yes (stop)	-	-	Yes CCP
	5d Cooling	Yes	No	No (stop)	-	No
	6b Freeze drying	Yes	No	No (stop)	-	No
	7 Grinding	Yes	No	No (stop)	-	No
	8 Packaging	Yes	No	Yes	No *	PrP
**PROCESS D**	1-2-3 Reception/Fasting/Sieving	Yes	No	Yes	No *	PrP
	4b Cold slaughtering	Yes	No	No (stop)	-	No
	6b Freeze drying	Yes	No	No (stop)	-	No
	7 Grinding	Yes	No	No (stop)	-	No
	8 Packaging	Yes	No	Yes	No *	PrP

* Answer to Q4 was considered “No” because subsequent calculations provided in Section 4.3 demonstrate that hot slaughtering and hot drying do not eliminate significant hazards to an acceptable level, if present in raw materials.

**Table A2:** Inactivation parameters of biological hazards selected in the “short list” under wet conditions (a_w_ > 0.9).

Biological Hazards	Thermal Inactivation Characteristics			
	T_Ref_ (°C)	D_Ref_ * (min)	z (°C) *	Reference
*B. cereus* (spore)	95	2	(8–12.5)	[105]
*C. botulinum* (spore)			10	[106]
Type I	121.1	0.21		
Type II	80	(0.6–1.25)		
Type III	104	(0.1–0.9)		
Type IV	104	(0.8–1.12)		
*C. perfringens* (spore)	100	(0.2–43)	(10.6–13.7)	[107]
*Cronobacter* spp.	60	(0.9–4.4)	5.6	[108]
STEC	60	(0.5–3)	(3.5–7)	[69]
*L. monocytogenes*	65	(0.2–2)	(4–11)	[109]
*Salmonella* spp.	60	(2–6)	6.5	[64]
*S. aureus*	60	(0.8–10)	7	[71]
toxin	121	(8.3–34)	(25–33)	[110]

* Values considered for calculation as a worst case, meaning the higher D_ref_ and z.

**Table A3:** Inactivation parameters of biological hazards selected in the “short list” under dried conditions (a_w_ < 0.6).

Biological Hazards	Thermal Inactivation Characteristics			
	T_Ref_ (°C)	D_Ref_ (min)	z (°C)	Reference
*Cronobacter* spp.	85	1.7	47	[108]
*E. coli*	85	1	31	[69]
*Salmonella* spp.	85	2.3	35	[64]

**Table A4:** Survival, growth, and toxin production characteristics of significant biological hazards.

Biological Hazards	T_min_ (°C)	T_opt_ (°C)	T_max_ (°C)	pH_min_	pH_opt_	pH_max_	a_w min_	a_w opt_	a_w max_	Reference
*B. cereus*	4	30–37	55	4.3	6–7	9.3	0.92	0.99–1		[105]
Toxinogenes	10	20–25	40							
*C. botulinum*										[106]
Group I	10	35–40	48	4.6		9	0.94		0.97	
Group II	2.5	18–25	45	5.1	6.1–6.3	9	0.97			
Toxinogenes	10						0.94			
*C. perfringens*	10	40–45	52	5	6–7	8.3	0.95/0.97	0.99		[107]
*Cronobacter* spp.	5.5	39	47	3.89	5–9	10				[108]
STEC *	6	40	45.5	4.4	6–7	9	0.95	0.995		[69]
*L. monocytogenes*	−2	30–37	45	4–4.3	7	9.6	0.92	0.99		[109]
*Salmonella* spp.	5	35–37	50	3.8	7–7.5	9.5	0.94	0.99		[64]
*S. aureus*	6	35–41	48	4	6–7	10	0.83	0.99	0.99	[71]
Toxinogenes	10	34–40	45	5	7–8	9.6	0.86	0.99	0.99	

* Values corresponds to *E. coli* O157:H7.
